# Motor Unit Discharge Variability Is Increased in Mild-To-Moderate Parkinson's Disease

**DOI:** 10.3389/fneur.2020.00477

**Published:** 2020-05-29

**Authors:** Jessica M. Wilson, Christopher K. Thompson, Laura Miller McPherson, Cindy Zadikoff, C.J. Heckman, Colum D. MacKinnon

**Affiliations:** ^1^Department of Physical Therapy and Human Movement Sciences, Northwestern University, Chicago, IL, United States; ^2^Department of Health and Rehabilitation Sciences, Temple University, Philadelphia, PA, United States; ^3^Program in Physical Therapy, Washington University School of Medicine, St. Louis, MO, United States; ^4^Department of Neurology, Washington University School of Medicine, St. Louis, MO, United States; ^5^Department of Neurology, Northwestern University, Chicago, IL, United States; ^6^Department of Physiology, Northwestern University, Chicago, IL, United States; ^7^Department of Physical Medicine and Rehabilitation, Northwestern University, Chicago, IL, United States; ^8^Department of Neurology, University of Minnesota, Minneapolis, MN, United States

**Keywords:** motor unit, Parkinson's disease, EMG, biceps brachii, triceps brachii

## Abstract

Individuals with Parkinson's disease (PD) demonstrate deficits in muscle activation such as decreased amplitude and inappropriate bursting. There is evidence that some of these disturbances are more pronounced in extensor vs. flexor muscles. Surface EMG has been used widely to quantify muscle activation deficits in PD, but analysis of discharge of the underlying motor units may provide greater insight and be more sensitive to changes early in the disease. Of the few studies that have examined motor unit discharge in PD, the majority were conducted in the first dorsal interosseous, and no studies have measured motor units from extensor and flexor muscles within the same cohort. The objective of this study was to characterize the firing behavior of single motor units in the elbow flexor and extensor muscles during isometric contractions in people with mild-to-moderate PD. Ten individuals with PD (off-medication) and nine healthy controls were tested. Motor unit spike times were recorded via intramuscular EMG from the biceps and triceps brachii muscles during 30-s isometric contractions at 10% maximum voluntary elbow flexion and elbow extension torque, respectively. We selected variables of mean motor unit discharge rate, discharge variability, and torque variability to evaluate motor abnormalities in the PD group. The effects of group, muscle, and group-by-muscle on each variable were determined using separate linear mixed models. Discharge rate and torque variability were not different between groups, but discharge variability was significantly higher in the PD group for both muscles combined (*p* < 0.0001). We also evaluated the asymmetry in these motor variables between the triceps and biceps for each individual participant with PD to evaluate whether there was an association with disease severity. The difference in torque variability between elbow flexion and extension was significantly correlated with both the Hoehn and Yahr scale (rho = 0.71) and UPDRS (rho = 0.62). Our findings demonstrate that variability in motor output, rather than decreased discharge rates, may contribute to motor dysfunction in people with mild-to-moderate PD. Our findings provide insight into altered neural control of movement in PD and demonstrate the importance of measuring from multiple muscles within the same cohort.

## Introduction

A hallmark of Parkinson's disease (PD) is the presence of an abnormal pattern of muscle activity when performing voluntary movements (1–4). This abnormal pattern is characterized by decreased EMG amplitude and the presence of multiple agonist bursts with highly variable duration rather than a single fused agonist burst. These changes in surface EMG patterns during voluntary movement are observed early in disease progression (4). Previous studies have provided evidence that abnormalities in muscle activation, and the accompanying deficits in motor output, are more pronounced in extensor muscles compared to flexor muscles. Ballistic elbow extension movements in people with PD are associated with increased slowing and more agonist bursts when compared with flexion movements (5, 6). Similarly, deficits in isometric force generation and movement velocity are greater in elbow extension than flexion, and these relative differences persist in both the off and on medication states (5–7). Greater deficits in extensor compared to flexor muscle function in PD have also been demonstrated in the lower extremity (5–10). These reductions in strength and movement speed have been ascribed to impairment in the ability to activate the agonist muscle rather than to co-contraction of agonist-antagonist muscles.

Surface EMG, which provides an interference signal of the electrical activity of its constituent motor units, has provided a substantial amount of information about abnormal muscle activation in PD. Yet, specific characteristics of the underlying motor unit discharge, such as discharge variability, cannot be extracted from the interference signal without its decomposition into individual motor unit spike trains (11). Further, individual motor unit discharge patterns may be more sensitive to disturbances of motor control and may thereby yield information that is not available with analysis of the interference EMG signal. Therefore, an investigation of motor unit discharge patterns may provide novel insight into the mechanisms of impaired muscle activation and asymmetry of extensor vs. flexor muscle function in PD. However, few studies have examined the activity of individual motor units in PD. The majority of those that have examined motor unit discharge were conducted in the first dorsal interosseous (FDI) muscle, and no studies have measured motor units from extensor and flexor muscles within the same cohort. Findings from these initial characterizations of motor unit firing abnormalities in PD include significantly lower firing rates in the finger extensors (12) and FDI (12–14), increases in firing rate variability in the FDI (12, 15, 16), alterations in recruitment order in the tibialis anterior (17), disturbances in rate modulation of the biceps brachii and FDI (18), and pauses in firing of the FDI, sometimes lasting up to 3 min in severe cases (14).

Given the differences in movement impairment between extensor and flexor muscles in PD and the paucity of studies with flexor and extensor recordings in the same person, the purpose of this study was to examine motor unit discharge rates and discharge variability in an elbow extensor (triceps brachii) and an elbow flexor (biceps brachii) in individuals with PD in the off-medication state compared with control individuals without PD. We chose to include individuals with mild-moderate motor impairment rather than more severe impairment due to the feasibility of testing them in the off-medication state. We hypothesized that discharge rates are decreased and that discharge variability is increased in individuals with PD compared to controls in the triceps but not the biceps. We also hypothesized that increases in discharge variability, if found, would be accompanied by increased torque variability, and decreases in discharge rates, if found, would be accompanied by decreases in maximal strength. Finally, we investigated in the PD group whether the postulated differences in discharge rate, discharge variability, and torque variability between muscle groups was associated with disease severity.

## Materials and Methods

Ten individuals with mild-to-moderate PD and nine age-matched control individuals without PD were included in the study. Participants with PD were included if they (8) had idiopathic PD with a tremor sub-score <2 according to the motor subsection (part III) of the UPDRS, (1) had no cognitive impairments [defined as Montreal Cognitive Assessment (MoCA) score > 26], (5) had no other previously known neurological disorders, (15) had no known injuries or other diseases that might interfere with motor function of the tested upper limb, and (12) were not currently on medications that may influence motor unit output such as selective serotonin reuptake inhibitors (SSRIs) or calcium channel blockers. Table 1 provides a summary of demographic information of the participants with PD. Nine of the 10 participants in this group were taking between one and three medications for PD-related symptoms (amantadine, ropinirole, levodopa/carbidopa, and/or pramipexole), and the remaining participant did not take any medication for PD. The control group consisted of seven men and two women with a mean ± SD age of 67.7 ± 6.2 years.

**Table 1:** Demographic information for participants with Parkinson's disease.

	**Age**	**Sex**	**Disease**	**Motor**	**Hoehn and**
	**(years)**		**duration**	**UPDRS**	**Yahr**
			**(years)**	**(off-medication)**	**(off-medication)**
Mean (SD)	64.4 (10.8)	8 M/2 F	6.9 (3.2)	19 (10)	2 (1)
Range	43–78		3–14	7–38	1–4

Participants with PD were tested in the practically defined “off” medication state that followed a 12-h period of withdrawal from their PD medications. The control participants had no known history of neurological disorders and no known injuries or diseases that might interfere with motor function of the tested upper limb. All participants were required to abstain from caffeine for 12-h before the experiment to remove any possible effects of caffeine on motoneuron function (19, 20). Motor testing as well as a clinical assessment of disease severity using the motor subsection (part III) of the UPDRS and classification according to the Hoehn and Yahr scale (21) was conducted in all participants (both PD and controls). Control participants showed no abnormalities on the UPDRS (score = 0). The dominant limb was tested for all participants with the exception of one control participant (due to an injured shoulder muscle) and one participant with PD (due to excessive tremor in the dominant limb). All participants provided written informed consent to participate in the study, which was approved by the Institutional Review Board of Northwestern University in accordance with the ethical standards stipulated by the 1964 Declaration of Helsinki for research involving human participants.

### Experimental Procedures

Participants were seated in a Biodex chair (Biodex Medical Systems, Shirley, NY) with their tested arm fixed in 75° shoulder abduction, 45° shoulder flexion (horizontal adduction from the frontal plane), 90° elbow flexion, 15° pronation, and a neutral wrist and finger posture (22, 23). The participant's shoulder and waist were secured to the chair with straps to minimize auxiliary movements of the trunk. The forearm and hand were encased in a fiberglass cast and coupled via a weight-bearing ring-mount interface to a six-degree-of-freedom load cell (Model 45E15A; JR3, Woodland, CA). Prior to the main experimental trials, participants were asked to generate maximal efforts in elbow flexion and in elbow extension. Maximum voluntary torque (MVT) was calculated as the average of three consecutive maximum torque values within 10% of one another without the last repetition being the greatest. Real-time visual feedback of elbow flexion/extension torque was given through a computer monitor in front of the apparatus, and participants were given vigorous verbal encouragement through the duration of the MVT measurements.

For experimental trials, participants completed isometric contractions in elbow flexion and in elbow extension at 10% MVT. A green circle on the computer screen represented real-time visual feedback of elbow flexion/extension torque, and a red circle target was displayed to represent 10% MVT. Participants completed the experimental task as follows. After 5-s of baseline measurements obtained while the participant was relaxed, each participant produced an isometric ramp-and-hold contraction at their own pace (typically ~5-s to reach the target torque) so that the green circle on the computer screen reached the red circle target. They maintained 10% MVT by holding the circle in the target for 30-s and then decreased torque production back to zero at their own pace. Participants completed 1–2 practice trials before data collection to familiarize themselves with the task. Between each experimental trial, participants were given a 1-min break, and they were asked to produce small brief contractions of the agonist and antagonist muscles to ensure quiescence of muscle activity before the start of the next trial (22). Trials were visually inspected for quality of torque production and discarded and repeated if necessary. Eight out of 111 total trials across participants were discarded because of issues during the data collection (e.g., contraction of the wrong muscle, appeared to fall asleep during the trial) or with post-processing (e.g., no motor units could be detected).

### Data Collection

Orthogonal forces and torques generated at the forearm-load cell interface were digitized at a sampling rate of 1,024 Hz and converted into elbow flexion and extension torques using custom MATLAB software employing a Jacobian-based algorithm (The Mathworks, Natick, MA). Torque measurements were smoothed using an acausal moving average filter with a 250 ms window.

Intramuscular EMG in the long head of the biceps and the lateral head of the triceps were recorded using custom bipolar fine-wire steel electrodes with 1 mm recording surfaces (221-28SS-730, Jari Electrode Supply, Gilroy, CA). Each bipolar unit had barb lengths for the two wires of 1 and 2.5 mm. Two electrodes were inserted into each muscle. The signals from each electrode were band-pass filtered (300–10,000 Hz) and amplified (x1-10k) (DAM50 Bio-Amplifier, World Precision Instruments, Sarasota, FL) before digitization at 10,240 Hz (EMG-USB2+, OT Bioelettronica, Inc., Torino, Italy). Because torque and intramuscular EMG signals were collected on separate computers, a brief synchronization pulse was generated at the beginning of each trial and recorded by both computers as a reference point for offline synchronization. Intramuscular EMG recordings were collected using OTBiolab software (version 1.7.4735.19, OT Bioelettronica, Inc., Torino, Italy).

### Data and Statistical Analysis

Intramuscular EMG recordings were imported into EMGlab software (24) for decomposition into single motor unit spike trains. Briefly, EMGlab is a freely available software package that uses a template matching algorithm to extract the discharge times of individual motor units from intramuscular EMG. We analyzed our data using EMGlab as follows. Intramuscular EMG data were high-pass filtered at 1 kHz. Templates of motor unit action potentials were created for each identified motor unit and were used to automatically identify discharge times of that motor unit during a sliding window of 5–10-s. The resulting automatic decomposition was manually inspected and corrected as necessary based on the residual intramuscular EMG signal, which reaches zero when discharges for all motor units have been accurately identified and the motor unit action potential templates have been subtracted from the original EMG signal. Following decomposition of the current segment, the sliding window was moved ~4-s ahead and the process was repeated until full decomposition of the entire trial was achieved. EMGlab is able to resolve superimpositions of multiple motor unit action potentials via this semi-automatic process. The discharge times for each motor unit were exported at 1 kHz for subsequent processing. A single trained operator decomposed the intramuscular EMG data. A second trained operator was consulted on difficult trials and reviewed the final decomposition.

Custom MATLAB software was used to analyze the torque data and motor unit spike trains for each trial. The first and last 10-s of torque and motor unit data corresponding to the initial and final baseline phases and ascending and descending phases of the contraction were removed to isolate 30-s of the steady contraction; this data was used for all subsequent analyses. Inter-spike intervals (ISIs) and the associated mean ISI were calculated for each motor unit spike train. Mean discharge rate (pps) for each motor unit spike train was calculated as the reciprocal of the mean ISI. To quantify discharge variability, the coefficient of variation of the ISI (CoV_ISI_) was calculated for each motor unit as the standard deviation of the ISI values divided by the mean of the ISI values, multiplied by 100. Torque variability was calculated similarly using torque data (CoV_torque_).

A linear mixed model was used to determine main effects of group (PD, control) and muscle (biceps, triceps) as well as the interaction effect of muscle-by-group on the dependent variable of discharge rate. Discharge rate values from all recorded motor units were used. Group and muscle were included in the model as fixed factors, with muscle also included as a repeated factor. Participant was included in the model as a random factor with a random intercept. A scaled identity covariance structure was assumed for random and repeated factors. The same statistical analysis was used with the dependent variable of CoV_ISI_.

For elbow flexion MVT, elbow extension MVT, elbow flexion CoV_torque_, and elbow extension CoV_torque_, the mean value was calculated for each participant across trials for use in group analyses. The Shapiro–Wilk test was used to assess whether these data were normally distributed. A 2 × 2 repeated measures ANOVA was used to determine main effects of group (PD, control) and torque direction (elbow flexion, elbow extension; repeated factor) as well as the torque direction-by-group interaction on the dependent variable of MVT. The same analysis was used for the dependent variable of CoV_torque_.

We determined whether participants with higher disease severity demonstrated a greater difference in motor unit discharge rate, discharge variability, and torque variability between the triceps and the biceps. For each participant, we calculated the difference for each variable as the mean value for the biceps subtracted from the mean value for the triceps. Given our hypothesis that the triceps would exhibit increased motor unit discharge variability, we expected to find a positive correlation between disease severity (Hoehn and Yahr, UPDRS) and the difference in CoV_ISI_ and CoV_torque_ across muscles (the hypothesized direction of correlation is based on the assumption that there is no difference in discharge variability between muscles in the control group). Because we hypothesized that the triceps would exhibit decreased motor unit discharge rates, we expected to find a negative correlation between disease severity and the difference in discharge rate across muscles. We calculated the Spearman correlation coefficient for each comparison along with the associated 1-sided *p*-value.

Statistical analyses were conducted in SPSS. Statistical significance was determined as *p* < 0.05. Cases for which 0.05 < *p* < 0.10 are presented.

## Results

### Motor Unit Discharge Rates and Variability

**Figure 1:**
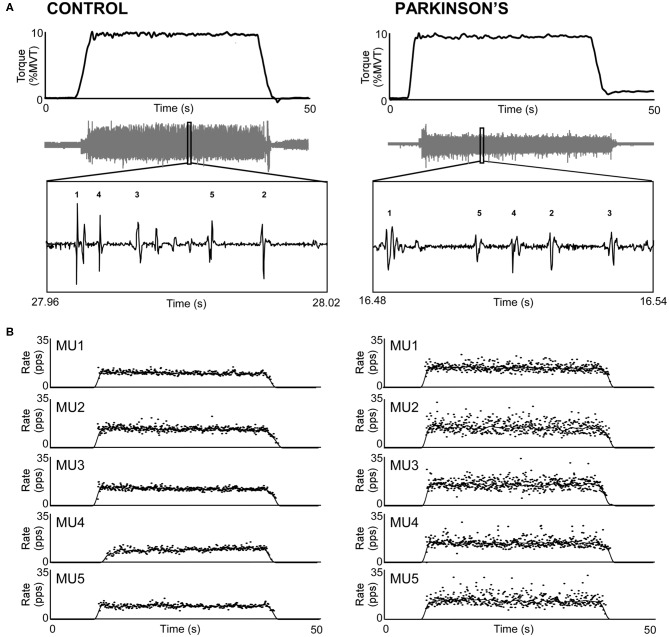
Sample trials from the triceps brachii of a control participant (left) and a participant with PD (right). **(A)** Participants produced 30-s isometric contractions at 10% MVT, and intramuscular EMG (gray trace) was decomposed into its constituent motor units. **(B)** Instantaneous discharge rate (pps) is shown for each motor unit labeled in A. Motor unit discharge rates are also shown smoothed with a 2-s Hanning window.

**Figure 2:**
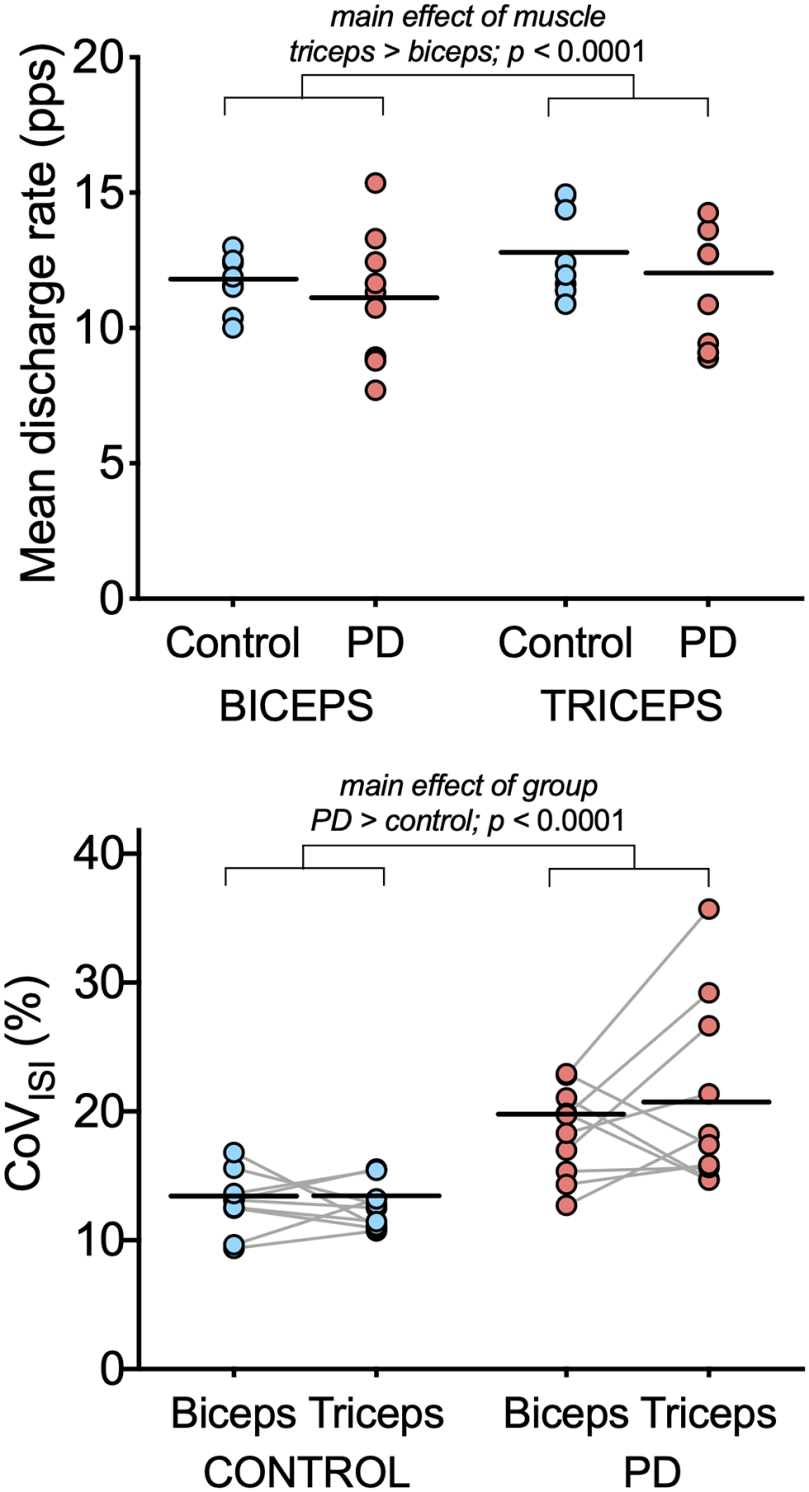
Motor unit discharge characteristics in biceps brachii and triceps brachii for the PD and control groups. Individual participant means (light blue circles for the control group and light red circles for the PD group) and group means (black horizontal bars) are shown for mean discharge rate (top) and CoV_ISI_ (bottom). Note that while individual participant means are shown for illustrative purposes, the linear mixed model to assess effects of group and muscle were computed using data from all motor units.

Figure 1 shows representative torque and triceps motor unit instantaneous discharge rates during a 10% MVT elbow extension trial from one control participant (left panel) and one participant with PD (right panel). A summary of individual participant and group mean discharge characteristics for biceps and triceps motor units for control and PD groups are shown in Table 2 and Figure 2. A total of 246 and 228 spike trains were analyzed in the PD and control groups, respectively.

**Table 2:** Summary of motor unit discharge characteristics.

**Muscle**	**Group**	**No. of MU** **per participant** **(mean ± SD)**	**Mean discharge** **rate (pps)** **(mean ± SE)**	**CoV_**ISI**_ (%)** **(mean ± SE)**
Biceps brachii	Control	12 ± 7	11.75 ± 0.56	12.64 ± 1.16
	PD	13 ± 7	11.07 ± 0.53	18.8 ± 1.07
Triceps brachii	Control	14 ± 8	12.76 ± 0.56	12.75 ± 1.13
	PD	12 ± 4	11.98 ± 0.53	19.67 ± 1.09

*Group mean ± SE values for discharge rate and CoV_ISI_ are displayed as estimates from the associated linear mixed model*.

Table 3 summarizes results of the linear mixed model used to determine the effects of group, muscle, and muscle-by-group on mean discharge rate and on CoV_ISI_. For mean discharge rate, the main effect of muscle was statistically significant, with higher mean discharge rates found in the triceps (mean ± SE: 11.41 ± 0.39 pps vs. 12.37 ± 0.39 for biceps and triceps, respectively; *p* < 0.0001). The main effect of group was not significant (11.53 ± 0.52 pps vs. 12.3 ± 0.55 pps for the PD and control groups, respectively; *p* = 0.34), nor was the muscle-by-group interaction (*p* = 0.78).

**Table 3:** Results from the linear mixed models testing the effects of muscle, group, and muscle-by-group on mean discharge rate and on CoV_ISI_.

	**Dependent variable**	
	**Mean discharge rate**	**CoV_**ISI**_**
**Fixed effects**		
Muscle	*p* < 0.0001	*p* = 0.45
	*F*_(1,458)_ = 28.4	*F*_(1,465)_ = 0.58
Group	*p* = 0.34	*p* < 0.0001
	*F*_(1,7)_ = 0.95	*F*_(1,16)_ = 20.5
Muscle by Group	*p* = 0.78	*p* = 0.56
	*F*_(1,458)_ = 0.1	*F*_(1,465)_ = 0.35

For CoV_ISI_, the main effect of muscle was not statistically significant (mean ± SE: 15.74 ± 0.79% vs. 16.21 ± 7.8% for biceps and triceps, respectively; *p* = 0.45), nor was the muscle-by-group interaction (*p* = 0.56). The main effect of group indicated significantly higher CoV_ISI_ values in the PD group than in the control group (19.26 ± 1.0% vs. 12.70 ± 1.05% for PD and control groups, respectively; *p* < 0.0001).

### Maximal Strength and Torque Variability

Maximum voluntary torque and CoV_ISI_ values were normally distributed for each group and torque direction (*p*-values ranging from 0.10 to 0.98). Maximum voluntary torque did not differ between the two groups [main effect of group: *F*_(1,7)_ = 0.14, *p* = 0.71; group × torque direction interaction: *F*_(1,7)_ = 1.34, *p* = 0.26]. In elbow flexion, the group mean ± SD MVT was 65.1 ± 25.7 N-m in the control group and 58.5 ± 14.4 N-m in the PD group. In elbow extension, the group mean ± SD MVT was 41.6 ± 16.7 N-m in the control group and 42.5 ±12.5 N-m in the PD group. MVT values were greater in elbow flexion than elbow extension for the groups combined [main effect of torque direction: *F*_(1,7)_ = 36.6, *p* < 0.0001].

**Figure 3:**
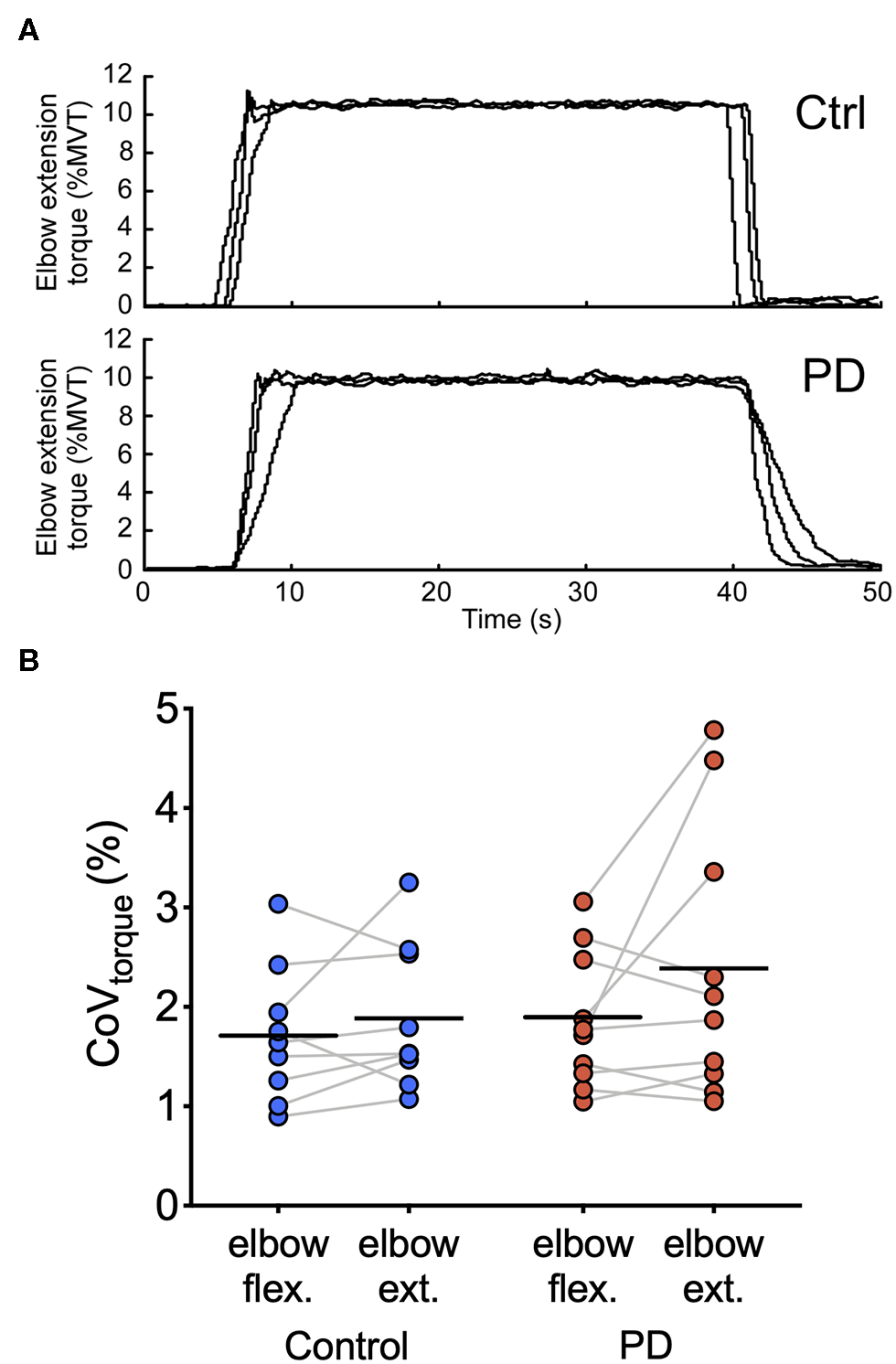
Torque traces and torque variability during steady contractions. **(A)** Torque traces at 10% elbow extension MVT from a control participant (top) and a participant with PD (bottom) are shown. Data from three separate trials are superimposed. **(B)** Individual participant means (dark blue circles for the control group and dark red circles for the PD group) and group means (black horizontal bars) are shown for CoV_torque_ for elbow flexion and elbow extension torque directions.

Figure 3 shows representative torque data and individual and group means for CoV_torque_ for each torque direction. In elbow flexion, the group mean ± SD CoV_torque_ was 1.7 ± 0.7% in the control group and 1.9 ± 0.7% in the PD group. In elbow extension, the group mean ± SD CoV_torque_ was 1.9 ± 0.7% in the control group and 2.4 ± 1.4 N-m in the PD group. CoV_torque_ did not differ between the two groups [main effect of group: *F*_(1,7)_ = 0.73, *p* = 0.40; group × torque direction interaction: *F*_(1,7)_ = 0.83, *p* = 0.38] or between torque directions [main effect of torque direction: *F*_(1,7)_ = 3.1, *p* = 0.098]. Visual inspection of the individual mean values shown in Figure 3 revealed that there were large increases in CoV_torque_ for elbow extension compared with elbow flexion for three of the 10 participants in the PD group compared with only one participant in the control group.

### Motor Variables and Disease Severity

**Figure 4:**
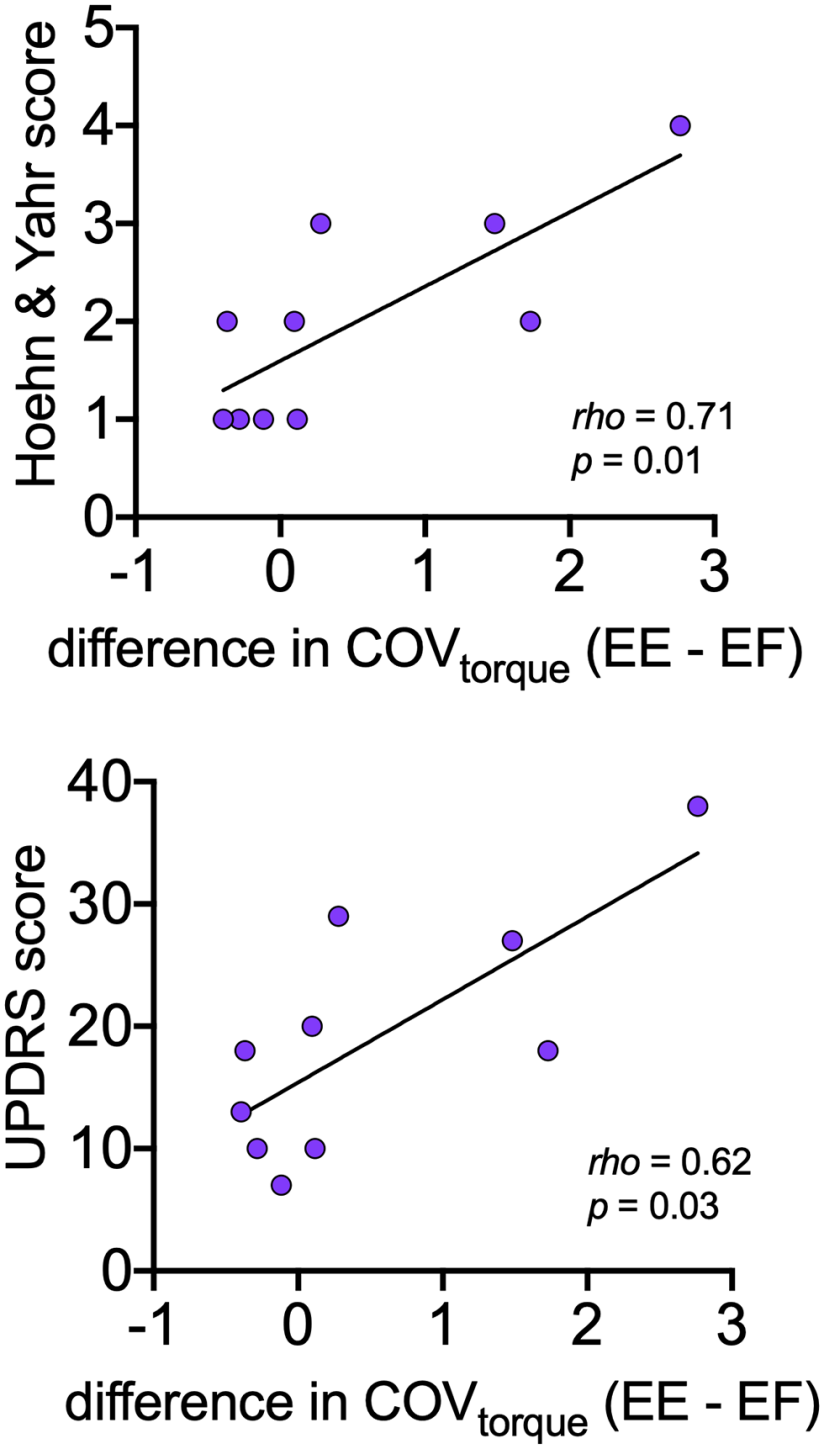
Relationships between asymmetry of torque variability between elbow extension and elbow flexion with disease severity (Hoehn and Yahr, UPDRS) within the PD group. For each participant, asymmetry is expressed as the mean CoV_torque_ for elbow flexion subtracted from the mean CoV_torque_ for elbow extension.

Table 4 presents the Spearman correlation coefficients and associated *p*-values for the comparisons between motor variables (CoV_ISI_, CoV_torque_, mean discharge rate) and disease severity (Hoehn and Yahr, UPDRS). CoV_torque_ was the only variable that was significantly correlated with disease severity, and it exhibited a moderate-strong correlation (Hoehn and Yahr: rho = 0.71, *p* = 0.01; UPDRS: rho = 0.62, *p* = 0.03). Figure 4 presents these correlations graphically.

**Table 4:** Correlations between triceps-biceps asymmetry of motor variables and disease severity.

	**H&Y**	**UPDRS**
Triceps-biceps difference in CoV_ISI_	rho = 0.04	rho = 0.11
	*p* = 0.46	*p =* 0.38
Triceps-biceps difference in mean discharge rate	rho = −0.13	rho = −0.23
	*p* = 0.36	*p =* 0.27
Elbow extension-flexion difference in CoV_torque_	rho = 0.71	rho = 0.62
	*p* = 0.01	*p* = 0.03

## Discussion

The present study investigated differences in motor unit discharge rate and discharge variability of an elbow flexor (biceps brachii) and an elbow extensor (triceps brachii) among individuals with mild-to-moderate PD and control participants without PD.

### Abnormalities in Motor Unit Behavior in Mild-to-Moderate Parkinson's Disease

Both Dietz et al. (12) and Milner-Brown et al. (14) observed significantly lower firing rates in individuals with severe motor symptoms of PD, with firing frequencies as low as 2–3 Hz and prolonged pauses in firing lasting from 10-s to 3-min (14). These significantly lower firing rates were present regardless of the presence of tremor. Dietz et al. (12) posited that these abnormally low discharge rates may be a common element of motoneuron function in PD. Such low discharge frequencies can contribute to variability in torque generation since muscle fiber twitches would remain unfused (13). However, in the present study, we did not observe any significant differences in mean discharge rate between our cohort with mild-to-moderate PD and control subjects in either muscle. This finding is consistent with the lack of strength differences observed between groups. A previous study showed differences in isometric strength between the elbow extensors and flexors in people with PD (5). This discrepancy might be explained by differences in the stage of disease between cohorts. It is possible that significant changes in slowing of motor unit discharge do not manifest in the earlier stages of PD but may be a source of increasing motor impairment with progression of the disease.

The main finding of this experiment was a significantly greater discharge variability in individuals with PD compared to controls in both the biceps and triceps muscles. Changes in discharge variability have also been reported in previous studies (12, 13). In particular, our results corroborate those of Dengler et al. (15), which showed that the first dorsal interosseous muscle of those with mild-to-moderate PD exhibited an increase in discharge rate variance without significant changes in mean discharge rates. In individuals without neurological disease, increased discharge variability is a known contributor to increased torque variability (25–28), but in the present study, torque variability was not significantly different between groups. A dissociation between discharge variability and torque variability has been documented in the literature before (29). The level of torque being used in this study may also be a contributing factor, as the presence of signal-dependent noise in the torque signal linearly increases with force output (30). It is possible that in the early stages of the disease, an insufficient part of the motoneuron pool is adversely affected enough for increased discharge variability to translate into a loss of force steadiness; the increased variance of CoV_ISI_ values within individuals with PD supports this possibility. The current results do suggest, however, that discharge variability may be a greater contributor to abnormal motor output than changes in discharge rates. Furthermore, results indicate that deficits in motor unit behavior may be observed in PD before the onset of deficits in torque generation.

### Extensor vs. Flexor Deficits in PD

Other investigators have demonstrated that individuals with PD have greater deficits in extensor compared with flexor function in both the lower and upper limbs, suggesting differential impairment of neural activation of the flexor and extensor muscles (5–10). Thus, we also sought to explore potential differences in motor unit firing abnormalities in PD between the biceps and triceps. Interestingly, discharge variability was increased in both biceps and triceps of individuals with PD compared to controls and further, the difference in torque variability between elbow extension and elbow flexion contractions was positively correlated with disease severity. In other words, for individuals with more severe PD, their elbow extension torque variability was greater than their elbow flexion torque variability and to a larger extent. These findings suggests that motor unit abnormalities in PD are not extensor-specific, at least in the case of discharge variability in a mild-moderate cohort; however, some asymmetry between muscle groups can be observed in terms of torque variability.

The participants with PD in the present study were studied in their OFF-medication state. Treatment with levodopa has been shown to significantly improve extensor force production more than the flexors in PD (5). This suggests that muscle asymmetry in PD is primarily mediated by the loss of dopamine in the striatum. Indeed, studies that have compared motor unit firing properties between the on and off medication states have shown that levodopa is associated with increased average firing rates and reduced discharge variability in the first dorsal interosseous (14, 18). Future work comparing motor unit discharge characteristics in the biceps and triceps of individuals with mild-to-moderate PD in the off-medication vs. the on-mediation state is warranted.

### Limitations and Clinical Implications

Our results demonstrate that abnormalities in spinal motor output are observable in people with mild-to-moderate PD, characterized by an increase in discharge variability but not a difference in discharge rate. In addition, our data suggest that deficits in motor unit output can be detected before some deficits in force generation. These findings should be considered when comparing populations with mild-to-moderate and severe PD and for the development of longitudinal studies and rehabilitation therapies.

There are several limitations to the present work that should be considered. Our study was cross-sectional in nature and isolated to the off-medication PD population with mild-moderate disease. Additional work should be conducted to explore how our measurements would change longitudinally, in the on-medication status, and in individuals with more severe disease. Additionally, our sample size is relatively small due to the challenges inherent to taking invasive recordings from an older population with neurological injury; nonetheless, our findings provide important insight into alterations in neural control that underlie the movement dysfunction that presents with PD.

## Data Availability Statement

The datasets generated for this study are available on request to the corresponding author.

## Ethics Statement

The presented study involving human participants was reviewed and approved by the Institutional Review Board of Northwestern University. The participants provided their written informed consent to participate in this study.

## Author Contributions

JW, CZ, CH, and CM conceived and designed research. JW and CZ recruited study participants. JW and CT performed experiments. JW, CT, and LM analyzed data. JW, CT, LM, CH, and CM interpreted results of experiments. JW, CT, and LM drafted the figures and manuscript, and all authors edited, revised, and approved final version of manuscript.

## Conflict of Interest

The authors declare that the research was conducted in the absence of any commercial or financial relationships that could be construed as a potential conflict of interest.
